# Effectiveness and safety of a simple home-based rehabilitation program in pulmonary arterial hypertension: an interventional pilot study

**DOI:** 10.1186/s13102-021-00315-y

**Published:** 2021-07-28

**Authors:** Mariusz Wojciuk, Mariusz Ciolkiewicz, Anna Kuryliszyn-Moskal, Sylwia Chwiesko-Minarowska, Emilia Sawicka, Katarzyna Ptaszynska-Kopczynska, Karol Kaminski

**Affiliations:** 1 Department of Rehabilitation, Medical University of Bialystok, ul. Sklodowska-Curie 24A, 15-089, Bialystok, Poland; 2 Department of Population Medicine and Lifestyle Diseases Prevention, Medical University of Bialystok, Bialystok, Poland; 3 Department of Cardiology, Medical University of Bialystok, Bialystok, Poland

**Keywords:** PAH, Exercise training, Cardiac rehabilitation, Respiratory training, Home-based exercise program

## Abstract

**Background:**

Rehabilitation plays an important role in the management of patients with pulmonary arterial hypertension (PAH) and current guidelines recommend implementation of a monitored individualized exercise training program as adjuvant therapy for stable PAH patients on optimal medical treatment. An optimal rehabilitation model for this group of patients has not yet been established. This randomized prospective study assessed the effectiveness and safety of a 6-month home-based caregiver-supervised rehabilitation program among patients with pulmonary arterial hypertension.

**Methods:**

A total of 39 patients with PAH were divided into two groups: intervention group (16 patients), subjected to a 6-month home-based physical training and respiratory rehabilitation program adapted to the clinical status of participants, and control group (23 patients) who did not perform physical training. The 6-min walk test (6MWT), measurement of respiratory muscle strength, quality of life assessment (SF-36, Fatigue Severity Scale – FSS) were performed before study commencement, and after 6 and 12 months. Adherence to exercise protocol and occurrence of adverse events were also assessed.

**Results:**

Physical training significantly improved 6MWT distance (by 71.38 ± 83.4 m after 6 months (*p* = 0.004), which remained increased after 12 months (*p* = 0.043), and respiratory muscle strength after 6 and 12 months (*p* < 0.01). Significant improvement in quality of life was observed after the training period with the use of the SF-36 questionnaire (Physical Functioning, *p* < 0.001; Role Physical, *p* = 0.015; Vitality, *p* = 0.022; Role Emotional, *p* = 0.029; Physical Component Summary, *p* = 0.005), but it did not persist after study completion. Adherence to exercise protocol was on average 91.88 ± 14.1%. No serious adverse events were noted.

**Conclusion:**

According to study results, the home-based rehabilitation program dedicated to PAH patients is safe and effective. It improves functional parameters and quality of life. Strength of respiratory muscles and 6MWD remain increased 6 months after training cessation.

**Trial registration:**

ClinicalTrials.gov, NCT03780803. Registered 12 December 2018

## Background

Pulmonary hypertension is defined as a mean pulmonary arterial pressure (mPAP) of 25 mmHg or greater at rest, confirmed by right-heart catheterization (RHC) [1]. Pulmonary arterial hypertension (PAH) is a rare chronic disease leading to decreased physical activity, right-sided heart failure and a reduced life expectancy. PAH is characterized by the presence of pre-capillary pulmonary hypertension and pulmonary vascular resistance > 3 Wood units, in the absence of other causes of pre-capillary pulmonary hypertension such as severe lung disease, chronic thromboembolism or other rare causes [1, 2]. Apart from hemodynamic alterations, including increased pulmonary resistance, decreased stroke volume and cardiac index, PAH also induces other pathologic changes such as reduced muscle strength and endurance, decreased inspiratory muscle strength, which result in decreased peak VO_2_, progressive fatigue, dyspnea and deterioration in exercise capacity [3].

The main goals of PAH therapy are to ensure an acceptable level of quality of life in the earliest stages of the disease, reduce disease progression, and improve patient prognosis [4, 5], which remains poor [6, 7]. The European Respiratory Society/European Cardiology Society (ERS/ESC) guidelines published in 2015 recommend the implementation of monitored individualized exercise training as adjuvant therapy for stable PAH patients on optimal medical treatment [8]. Since then, data regarding the effect of physical activity on disease progression and prognosis have been published [9–16]. Regrettably, a clinically useful definition of exercise for pulmonary hypertension is still not available [17], and diversity in training forms and models highlights the necessity for more precise analysis.

According to the ERS statement on exercise training and rehabilitation in patients with severe chronic pulmonary hypertension [9], there is a pressing need to develop specialized rehabilitation programs, and to identify the most beneficial training models in further studies. To date, no optimal rehabilitation program striking the right balance between potential benefits and possible risks has been designed. Analyses in the available literature, which confirm the positive impact of regular exercise training not only on muscle function, exercise capacity and patient quality of life, but also pulmonary hemodynamics and right ventricular function, concern inpatient and outpatient rehabilitation models. An inadequate number of studies exploring the home-based rehabilitation model for patients with PAH have been published to date.

The principal aim of the present study was to evaluate the safety and effect of a 6-month home-based rehabilitation program on the quality of life, symptoms, physical endurance, strength of respiratory and skeletal muscles, and body mass composition of patients with PAH. The secondary aim of the study was to investigate the effects of therapy after six months of exercise cessation.

## Methods

The present prospective interventional pilot study was conducted between March 2019 and July 2020 at the Rehabilitation Department of the Medical University of Bialystok, Poland. Study participants were patients of the Cardiology Outpatient Clinic and the Department of Cardiology of the University Hospital in Bialystok. The study was conducted in accordance with the Declaration of Helsinki Principles for Medical Research Involving Human Subjects and was approved by an independent local bioethics committee. The study protocol was registered at ClinicalTrials.gov and was assigned the number NCT03780803. Both verbal and written consent for study participation was obtained from each participant prior to the commencement of the study. All subjects were informed of the objectives, procedures, and potential risks or discomfort associated with study participation.

A total of 46 patients with a diagnosis of PAH (Group 1 in the comprehensive clinical classification of pulmonary hypertension according to the ERS/ESC guidelines [8]) based on right-heart catheterization (mean pulmonary artery pressure (mPAP) ≥25 mmHg, pulmonary artery wedge pressure (PAWP) ≤15 mmHg) were enrolled in the study. All patients were in the World Health Organization Functional Class II or III (WHO-FC) and stable during the last three months of the study. Echocardiography, a perfusion lung scan, respiratory function tests and computed tomography were performed prior to the initiation of specific treatment to rule out other causes of pulmonary hypertension. During the period of study there were no changes in specific PAH therapy.

Exclusion criteria were as follows: significant modification in the treatment of the main disease within three months of study commencement, severe lung disease, such as chronic obstructive pulmonary disease or asthma, loss of consciousness within three months of study commencement, active neoplastic disease with poor prognosis, acute inflammation up to four weeks before enrollment, anemia (Hgb < 11 g/dl – male, < 10 g/dl – female), electrolyte and hormonal disorders within four weeks before enrollment, other clinical situations that made it impossible for the patient to participate in the rehabilitation program, and contraindications in accordance with the applicable standards of Comprehensive Cardiac Rehabilitation. Individuals with intellectual disability were also excluded from the study.

**Fig. 1:**
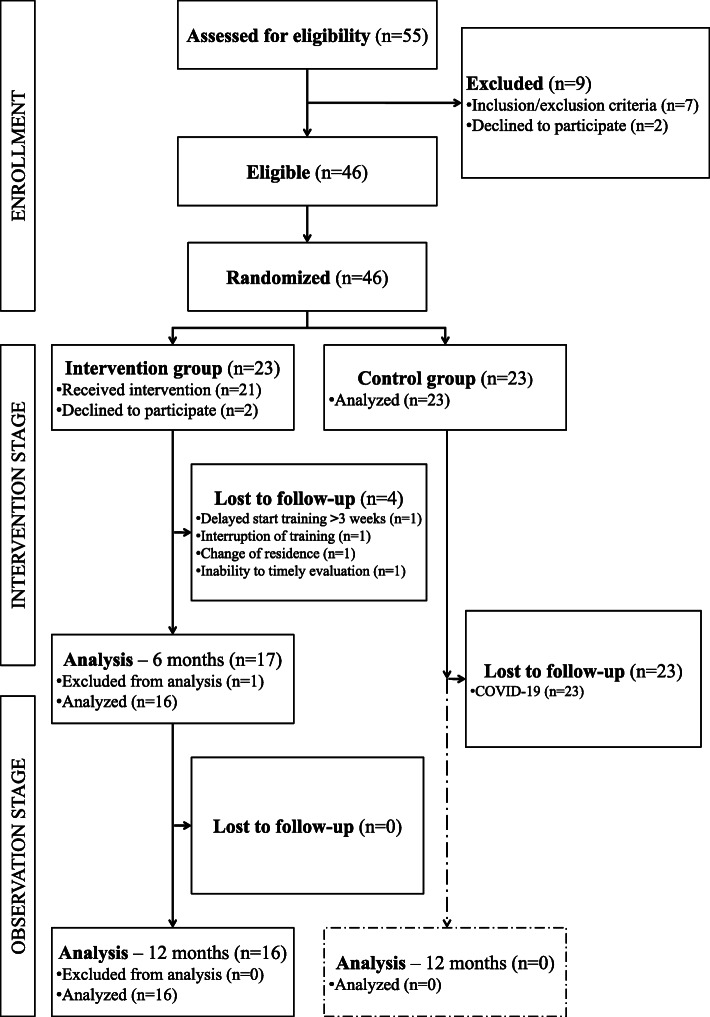
Diagram showing the flow of participants through the study

Patients were randomly assigned to either the intervention or the control group (1:1). Randomization was performed using Research Randomizer (Version 4.0) by an investigator who was not involved in the assessment of study outcomes. Figure 1 shows the flow of participants through the study. Not all participants were included in the final analysis. This was due to withdrawal of consent for participation in the study (*n* = 2), failure to start training within three weeks of receiving instruction (*n* = 1), termination of training in its initial phase (*n* = 1), change of residence (*n* = 1), and inability to attend scheduled assessments (*n* = 1). Most of the data for one participant were lost. Subjects from the control group were not assessed because of reluctance to attend scheduled appointments, which was associated with the COVID-19 pandemic.

Participants had a full medical history and anthropometric measures taken, and clinical examination performed. Baseline WHO-FC was assessed. As part of the clinical evaluation, right-heart catheterization (RHC), transthoracic echocardiography (TTE), cardiopulmonary exercise testing (CPET), the 6-min walk test (6MWT), measurements of respiratory muscles and handgrip strength, and body composition analysis using bioimpedance analysis (BIA) were performed. Blood samples were collected for the following tests: a complete blood count, concentrations of N-terminal brain natriuretic peptide (NT-proBNP), and serum creatinine concentrations. Quality of life was assessed with the 36-Item Short Form Health Survey (SF-36) and Fatigue Severity Scale (FSS).

Right-heart catheterization (RHC) was performed in the study group with a standard technique using a balloon-tipped 7F Swan-Ganz catheter; cardiac output was measured by thermodilution method; pulmonary vascular resistance (PVR) was calculated with the formula PVR = (mPAP-PAWP)/cardiac output (CO) and expressed in Wood units (WU).

Transthoracic echocardiography (TTE) was performed in accordance with the guidelines published by the American Society of Echocardiography [18, 19] using the iE33x Matrix (Philips) echocardiograph.

Cardiopulmonary exercise testing (CPET) was conducted using Schiller Cardiovit CS-200 ErgoSpiro system (Schiller AG, Baar, Switzerland) according to the standard treadmill ramp protocol in accordance with guidelines published by the American Thoracic Society and American College of Chest Physicians [20]. A symptom-limited exercise treadmill test was performed. The speed and incline were increased gradually every 20 s. Patients were monitored three minutes before, during the test, and three minutes after exercise (electrocardiography, pulse oximetry). Blood pressure was measured prior to and following the test. Gas analysis was also performed during the test. Parameters such as peak oxygen consumption (peak VO_2_, ml/kg/min), exercise ventilatory efficiency defined as the slope of the linear relationship between ventilation and carbon dioxide output (VE/VCO_2_ slope), and anaerobic threshold (AT) were assessed.

The 6-min walk test (6 MWT) was performed in accordance with standardized guidelines of the American Thoracic Society (ATS) [21] along a 30-m corridor after a minimum of 10 min of rest. Prior to and following the test, blood pressure and heart rate were measured, dyspnea and fatigue were assessed using the 10-point Borg Scale. Blood oxygen saturation was measured with a pulse oximeter (OxyWatch C29) before the test, and in the second, fourth, and sixth minute of the test. The test and the post-exercise recovery phase were supervised by a physician. All measurements were obtained in the same conditions (primary outcome).

Hand grip strength (HGS) was measured in accordance with the recommendations of the American Society of Exercise Physiologists (ASEP) [22] using the Jamar hand dynamometer (Saehan DHD-1 Digital Hand Dynamometer). The patient was sitting upright in a chair without armrests, feet resting flat on the floor, arms adducted to the trunk, elbow joints at 90 degrees of flexion, forearms in neutral position, wrists at between 0 and 30 degrees of extension. Isometric contraction lasted five seconds, with a transitional period of one second at the beginning of the contraction. Three attempts were made for each side alternately, with a minimum of a one-minute break between repetitions. The best result was recorded.

The strength and function of respiratory muscles were measured using the MicroRPM Respiratory Pressure Meter (Micromedical Ltd., Rochester, Kent, UK) which measures maximum inspiratory pressure (PImax), and maximum expiratory pressure (PEmax) generated during forced inspiration and expiration. Measurements were performed in accordance with guidelines of the American Thoracic Society/European Respiratory Society (ATS/ERS) [23, 24]. Patients were instructed to insert a mouthpiece into the mouth, ensuring the flange was positioned over the gums and inside the lips. During the measurement of maximum inspiratory pressure, the patient exhaled to residual volume, emptying the lungs, and then performed the Mueller maneuver, a forced inhalation against the MicroRPM with as much effort as possible for as long as possible (minimum two seconds). The result was maximum average inspiratory pressure sustained over the first second of the test in centimeters of water (cmH_2_O). In order to measure maximum expiratory pressure, the patient inhaled to total lung capacity and performed the Valsalva maneuver, a forced exhalation against the MicroRPM with as much effort as possible for as long as possible (minimum 2 s). The result was maximum average expiratory pressure sustained over the first second of the test in cmH_2_O. The best result out of three repeated attempts was recorded.

Body composition analysis (bioelectrical impedance analysis – BIA) was performed using the In-Body (720) body composition analyzer (Biospace Co. Ltd., Seoul, Korea) in accordance with the manufacturer’s instructions. The palms of the patient’s hands and the soles of their feet were moistened prior to contact with the platform of the analyzer. Participants were instructed to stand straight, hold the handles of the analyzer and keep still, enabling constant contact with electrodes during the analysis.

The reference group consisted of 25 healthy individuals without cardiovascular diseases or other significant chronic diseases, including chronic obstructive pulmonary disease, asthma, rheumatoid arthritis, systemic sclerosis, chronic kidney and hepatic diseases, cancer and other medical conditions that would influence the results of the clinical examination. Participants had a full medical history taken, and clinical examination performed. Anthropometric measures, measurements of respiratory muscle and handgrip strength, and body composition analysis using BIA were also performed. Quality of life was assessed with the SF-36 and the FSS questionnaires. The group was needed mainly as a reference for measuring body composition, handgrip and respiratory muscle strength since there are no clearly established norms for these parameters in the Polish population.

### Intervention stage

#### Exercise training

A home training and respiratory rehabilitation program was developed and adapted according to the clinical status of study participants, who were provided with a booklet containing detailed descriptions and photographs of the exercises and recommendations regarding the training. In order to ensure safety during exercise, the intensity of physical effort was set at the level of 4–5 (fairly hard – hard) points on the 10-point Borg Scale and the training heart rate was at the level of 60–70% of heart rate reserve based on baseline parameters from CPET (heart rate reserve = maximum exercise heart rate – resting heart rate, training heart rate = resting heart rate + 60–70% of heart rate reserve). Training was performed once a day, at least 5 times per week, for 6 months (24 weeks). The optimal target training duration of 45–60 min (no less than 30 min) was reached within the first two weeks of starting the program. Training progression was expressed in parameters such as an increased number of repetitions of particular exercises, increased resistance during respiratory muscle training, duration of a training session, marching pace and distance covered. Patients were advised to be assisted during training sessions by a family member or a caregiver.

Each training session started with a warm-up (5–10 min) in order to prepare the cardiovascular and the locomotor systems. It consisted of 15 exercises of all body parts including resistance training of inspiratory muscles with the use of the Pulmogain, RespiTrain (Moretti, Italy) exercise apparatus. The device provided three-stage progression in training intensity. The patient, by inhaling deeply, tried to lift all the balls constituting resistance. Inspiration had to be maintained for as long as possible. The main part of the training effort was interval march training (lasting a minimum of 30 min), interrupted every two minutes by one of five respiratory exercises performed in the recommended order. Interval aerobic march training together with respiratory exercises in the proposed combination were implemented in order to decrease dyspnea and fatigue, improve exercise tolerance, decrease work of breathing, correct the breathing pattern, increase chest and diaphragm mobility, strengthen respiratory muscles and increase lung capacity. The training session ended with a cool-down (5–15 min) in order to prevent a sudden drop in blood pressure and heart rate.

Prior to starting the training program, each patient was shown how to perform the exercises properly, was familiarized with recommendations regarding physical activity, specific contraindications to training, and instructions on how to manage adverse effects during or after exercise. The significance of regular, monitored physical activity in cardiac rehabilitation in relation to the pathophysiology of the disease was emphasized. Each patient in the intervention group was provided with the Pulmogain, RespiTrain exercise apparatus and instructed in its usage. Patient instruction took place in the Department of Rehabilitation, University Hospital, Medical University of Bialystok, Bialystok, Poland.

#### Self-monitoring

Members of the intervention group were provided with pedometers (Pearly 3D, Spokey) to measure their daily physical activity. They were advised to wear the device in accordance with manufacturer’s instructions in the pelvic area from morning to night (removing it before a shower or bath). Every patient received a self-control diary in which data relating to each day of the intervention period were collected. Participants were asked to report the following before and after each training session: heart rate and blood pressure, dyspnea and fatigue on the 10-point Borg Scale, duration of the training session, the number of steps taken during the training session indicated by the pedometer, and the total number of steps taken per day (on training and non-training days) to allow for assessment of general activity levels of the participants. Patients were instructed to record all changes in symptoms, or situations which may have impacted the quality of performed exercises as well as adverse reactions not associated with training.

Patients from the intervention group had appointments in the Department of Rehabilitation one month and three months after starting the training program in order for the research team to check if the exercises were performed properly, to monitor diary completion, and to make potential modifications to the training program. The participants were supervised and supported via telephone (at least once a week). In addition, attending physicians had an opportunity to analyze patients’ self-control diaries during scheduled appointments in the Cardiology Outpatient Clinic. The control group was not subjected to any of the above-mentioned interventions.

#### Adherence

Regularity of exercise in cardiac rehabilitation plays an important role in the final outcome of a training program undertaken by the patient. Therefore, in the present study, repeated weekly telephone calls were made to verify adherence to the minimum recommended five training sessions per week. Moreover, the participants reported on their compliance during scheduled appointments in the Cardiology Outpatient Clinic, scheduled hospitalizations and follow-up appointments in the Rehabilitation Department. Adherence was defined as a percentage of training sessions performed per week over the period of six months of intervention (minimum 120 sessions in a 24-week period).

### Observation stage

Re-evaluation of patients from the intervention group was conducted immediately after cessation of the 6-month intervention, and six months later. During the observational phase, patients decided independently about continuation or discontinuation of the recommended training program and intensity of exercises. Those who decided to continue the training program were offered the support of the research team. Follow-up investigations included a medical history, physical examination, anthropometric measurements, assessment of WHO-FC, 6MWT, measurements of respiratory muscle and handgrip strength, body composition analysis (BIA), and completed SF-36 and FSS questionnaires.

### Statistical analysis

Data analysis was performed with use of statistical environment R 3.6.2 and additional packages dedicated to particular types of analysis and data visualization. For comparisons of multiple independent means (intergroup comparisons), Welch’s ANOVA was performed based on type 2 sum of squares. The Kruskall-Wallis test was used as the non-parametric equivalent of Welch’s ANOVA. For comparisons of multiple dependent means (comparisons of the effects of intervention in the study group), a one-way analysis of variance for repeated measures was used. If the assumption of sphericity was violated, tested with the Mauchly test, the Greenhouse-Geisser correction was applied. The Friedmann test was used as the nonparametric equivalent of this analysis. Strength of effect for analyzes of variance was measured using the eta squared. The Bonferroni correction was used for post-hoc analysis. For comparisons of two independent groups, Welch’s t tests not assuming homogeneity of variance were used. As the nonparametric equivalent of this test, the Mann-Whitney test was applied. The t-test for paired data and the non-parametric Wilcoxon test were used to compare two paired groups. Measurement of the effect size for t-tests was Cohen’s d, and for non-parametric tests, Pearson’s r. When comparisons of two groups were performed as post-hoc analyses, *p*-values were corrected using the Bonferroni correction for multiple comparisons. *P*-values < 0.05 were considered statistically significant.

## Results

Prior to the commencement of the rehabilitation program, there were statistically significant differences between the intervention group and the healthy reference group in parameters such as PImax (49.62 ± 20.89 and 81.96 ± 27.9 cmH_2_O, respectively, *p* < 0.001) and PEmax (76 ± 32.77 and 113.16 ± 36.55 cmH_2_O, respectively, *p* < 0.001), measurement of respiratory muscle strength, and scores of SF-36 (PCS: 40.11 ± 11.35 and 51.5 ± 7, respectively, *p* = 0.008) and FSS questionnaires (4.08 ± 1.6 and 1.87 ± 1.07, respectively, *p* < 0.001). There were no statistically significant differences in handgrip strength and body mass composition between PAH patients and healthy individuals.

Patients with PAH who completed the exercise training program formed the intervention group (16 individuals, age 48.9 ± 18.6 years, 7 female (44%), 9 male (56%)) and the control group (23 individuals, age 53.7 ± 12.8 years, 13 female (57%), 10 male (43%)). Baseline demographic and anthropometric data, functional parameters including the 6-min walk distance (6MWD), measurements of respiratory muscles and handgrip strength, body composition analysis, and assessment of quality of life after random allocation of patients to the intervention and control groups are summarized in Table 1. Presented data also includes the etiology of PAH, WHO-FC of PAH, comorbidities, specific PAH therapy, the RHC, TTE, CPET parameters and laboratory test results prior to intervention. Statistically significant differences were found only for the tricuspid annular plane systolic excursion (TAPSE), and NT-proBNP parameters. In the control group, the mean values of both parameters were significantly higher than in the intervention group (Table 1).

**Table 1:** Participants characteristics after randomly assigned to intervention group and control group

	intervention group	control group	p-value
Patients, n	16	23	
Age, years	48.88 ± 18.25	53.65 ± 12.79	NS
Female sex, % (n)	43.8 (7)	56.5 (13)	NS
Height, cm	170.62 ± 7.82	164.99 ± 8.89	NS
Weight, kg	75.29 ± 19.1	75.43 ± 15.71	NS
Body Mass Index, kg/m^2^	25.79 ± 5.78	27.8 ± 6.04	NS
**PAH etiology**			
Idiopathic, % (n)	37.5 (6)	52.2 (12)	NS
Connective tissue diseases, % (n)	6.3 (1)	13 (3)	
Congenital heart disease, % (n)	50 (8)	30.4 (7)	
Heritable, % (n)	6.3 (1)	4.4 (1)	
**WHO-FC**			
II, % (n)	50 (8)	61 (14)	NS
III, % (n)	50 (8)	39 (9)	
**Co-morbidities**			
Hypertension, % (n)	31.3 (5)	34.8 (8)	NS
Diabetes mellitus, % (n)	6.3 (1)	13 (3)	NS
Hyperlipidemia, % (n)	25 (4)	26.1 (6)	NS
Atrial fibrillation, % (n)	18.8 (3)	17.4 (4)	NS
**PAH - specific therapy**			
PDE5i, % (n)	50 (8)	30.4 (7)	NS
ERA, % (n)	18.8 (3)	4.4 (1)	
Prostanoid, % (n)	0 (0)	0 (0)	
PDE5i + ERA, % (n)	31.3 (5)	43.5 (10)	
PDE5i + Prostanoid, % (n)	0 (0)	17.4 (4)	
PDE5i + ERA + CCB, % (n)	0 (0)	4.4 (1)	
**Functional parameters**			
PImax, cmH_2_O	45.31 ± 23.82	52.61 ± 18.55	NS
PEmax, cmH_2_O	70.94 ± 32.75	79.52 ± 33.05	NS
Dominant hand grip strength, kg	37.67 ± 12.32	35.96 ± 15.22	NS
Non-dominant hand grip strength, kg	36.2 ± 14.35	35.14 ± 14.71	NS
6MWD, m	436.81 ± 81.69	444.83 ± 87.98	NS
METs	4.77 ± 1.38	3.95 ± 1.05	NS
WorkLoad in AT, W	108.29 ± 51.99	97 ± 36.42	NS
WorkLoad max, W	181.64 ± 80.41	149.23 ± 61.97	NS
HR max	134.21 ± 26.73	133.54 ± 19.84	NS
Peak VO_2_ in AT, ml/kg/min	11.74 ± 4.46	10.53 ± 3.91	NS
Peak VO_2_, ml/kg/min	16.66 ± 4.78	14.14 ± 4.22	NS
VCO_2_	1.24 ± 0.45	1.11 ± 0.47	NS
VE, l/min	52.84 ± 14.58	48.21 ± 17.71	NS
VE/VO_2_ slope	47.64 ± 17.74	41.96 ± 10.58	NS
VE/VCO_2_ slope	44.45 ± 14.51	43.11 ± 11.88	NS
**Echocardiography**			
RV basal diameter, cm	4.51 ± 0.68	4.25 ± 0.72	NS
RAA, cm^2^	24.11 ± 9.43	23.84 ± 7.48	NS
TAPSE, mm	17.47 ± 2.8	20.62 ± 4.27	**0.012**
IVC diameter, mm	16.75 ± 3.19	17.42 ± 3.56	NS
**Hemodynamics (RHC)**			
mPAP, mmHG	47.23 ± 14.86	45.84 ± 16.22	NS
PAWP, mmHG	11.08 ± 2.47	10.11 ± 2.53	NS
CO, l/min	4.95 ± 1.15	5.1 ± 1.18	NS
CI, l/min/m^2^	2.67 ± 0.6	2.76 ± 0.58	NS
PVR, Wood Units	7.55 ± 3.81	7.61 ± 4.49	NS
**Laboratory tests**			
NT-proBNP, pg/ml	190.83 ± 279.39	424.57 ± 412.23	**0.048**
Creatinine, mg/dl	0.95 ± 0.23	0.94 ± 0.16	NS
**Body Mass Composition**			
Percentage of Body Fat, %	31.32 ± 9.08	32.33 ± 10.56	NS
Waist-Hip Ratio (WHR)	0.96 ± 0.12	0.93 ± 0.1	NS
Skeletal Muscle Mass, kg	27.54 ± 6.43	27.01 ± 6	NS
Body Fat Mass, kg	24.54 ± 11.17	24.3 ± 10.03	NS
Arm Circumference	32.28 ± 5.47	32.1 ± 4.3	NS
Arm Muscle Circumference	25.29 ± 4.11	25.76 ± 2.98	NS
**SF-36**			
Physical Component Summary	40.11 ± 11.35	41.33 ± 8.45	NS
Mental Component Summary	47.55 ± 13.33	46.3 ± 9.55	NS
**Fatigue Severity Scale**	4.08 ± 1.6	4.03 ± 1.75	NS

Values are mean ± standard deviation

6MWD: 6-min walk distance; AT: anaerobic threshold; CCB: calcium channel blocker; CI: cardiac index; CO: cardiac output; ERA: endothelin receptor antagonist; HR: heart rate; IVC: inferior vena cava; MET: metabolic equivalent; mPAP: mean pulmonary arterial pressure; NS: not significant; PAH: pulmonary arterial hypertension; PAWP: pulmonary artery wedge pressure; PDE5i: phosphodiesterase type 5 inhibitor; PEmax: maximal expiratory pressure; PImax: maximal inspiratory pressure; PVR: pulmonary vascular resistance; RAA: right atrial area; RV: right ventricle; SF-36: 36-Item Short Form Health Survey; TAPSE: tricuspid annular plane systolic excursion; VCO_2_: carbon dioxide production; VE: ventilation; VE/VCO_2_: ventilatory equivalent for carbon dioxide; VE/VO_2_: ventilatory equivalent for oxygen; VO_2_: oxygen consumption; WHO-FC: world health organization - functional class

**Fig. 2:**
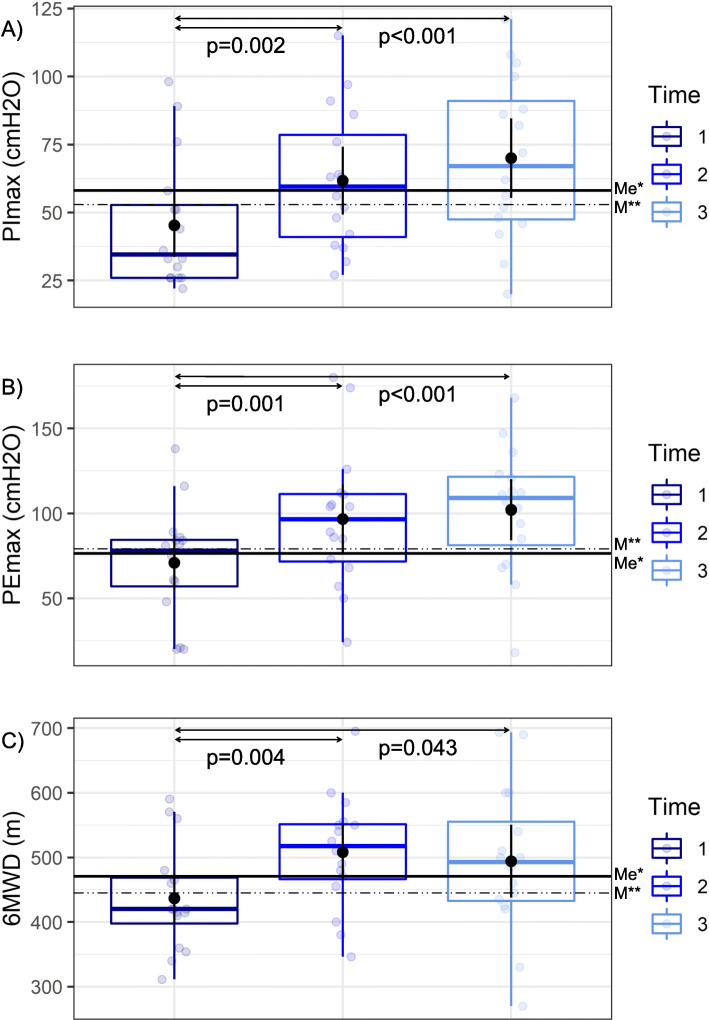
Effect of home-based exercise training on functional parameters. **A**) PImax: maximal inspiratory pressure; **B**) PEmax: maximal expiratory pressure; **C**) 6MWD: 6-min walk distance. The box contains the middle 50% of the data (interquartile range); the bold line inside the box indicates the median. The boxes were also marked with means with 95% confidence intervals represented by points and vertical lines. Time 1: measurements before intervention period; time 2: measurements after intervention period; time 3: measurements after observation period; Me*: median for the control group; M**: mean for the control group.

Data illustrating the effects of the intervention, divided according to intervals at which the measurements were taken are presented in Table 2. Physical training produced a statistically significant improvement in respiratory muscle strength, measured by PImax (Table 2, Fig. 2A) and PEmax (Table 2, Fig. 2B) after the 6-month intervention period (PImax: 16.38 ± 17.85 cmH_2_O, *p* = 0.002 and PEmax: 25.81 ± 24.58 cmH_2_O, *p* = 0.001), and after the observation period, within one year of patient inclusion in the study (PImax: 24.62 ± 19.09 cmH_2_O, *p* < 0.001 and PEmax: 31.19 ± 26.28 cmH_2_O, *p* < 0.001, Table 2). In post hoc analysis, a significant effect of intervals at which the measurements were taken was noted (*p* < 0.001). Statistically significant improvements in arm muscle circumference (0.43 ± 0.53, *p* = 0.005) and handgrip strength for both extremities (dominant: 5.67 ± 8.12 kg, *p* = 0.017 and non-dominant: 4.47 ± 6.22 kg, *p* = 0.015) after the intervention period were noted (Table 2). These parameters returned to baseline levels after training discontinuation during the observation period (Table 2). In post hoc analysis, a significant effect of intervals at which the measurements were taken noted (*p* < 0.05). A statistically significant improvement of 71.38 ± 83.4 m in the 6MWT distance was obtained(*p* = 0.004) which was maintained at the level of 57.12 ± 103.31 m (*p* = 0.043) after the observation period in comparison to the baseline value (Table 2, Fig. 2C). In post hoc analysis, a significant effect of intervals at which the measurements were taken was noted (*p* = 0.005). The level of oxygen saturation (SaO_2_) before 6MWT was statistically significantly higher after the intervention period (*p* = 0.029), and returned to initial values after the observation period (Table 2). Following the intervention phase and the observation period, study participants reported decreased dyspnea before 6MWT (*p* = 0.011 and *p* = 0.006, respectively, Table 2). The statistically significant improvement in quality of life achieved after the completion of the exercise training program measured using the SF-36 questionnaire (Physical Functioning, *p* < 0.001; Role Physical, *p* = 0.015; Vitality, *p* = 0.022; Role Emotional, *p* = 0.029; Physical Component Summary, *p* = 0.005) did not persist beyond completion of the study. Results of the one-way analysis of variance for repeated measurements of the evaluated parameters are presented in Table 2. No statistically significant changes in WHO-FC were observed.

**Table 2:** Training effects

	Baseline (t1)	time 1 → 2		Post-intervention (t2)	time 2 → 3		Post-observation (t3)	time 1 → 3	
		change	p-value		change	p-value		change	p-value
**Functional parameters**									
PImax, cmH_2_O	45.31 ± 23.82	**16.38**	**0.002**	61.69 ± 25.44	**8.25**	**0.01**	69.94 ± 29.83	**24.62**	**< 0.001**
PEmax, cmH_2_O	70.94 ± 32.75	**25.81**	**0.001**	96.75 ± 40.93	5.38	NS	102.12 ± 36.69	**31.19**	**< 0.001**
Dominant hand grip strength, kg	37.67 ± 12.32	**5.67**	**0.017**	43.33 ± 13.58	−1.8	NS	41.53 ± 15.81	3.87	NS
Nondominant hand grip strength, kg	36.2 ± 14.35	**4.47**	**0.015**	40.67 ± 13.95	**−4.33**	**0.003**	36.33 ± 14.21	0.13	NS
6MWD, m	436.81 ± 81.69	**71.38**	**0.004**	508.19 ± 87.97	−14.25	NS	493.94 ± 114.71	**57.12**	**0.043**
SaO_2_, % (before 6MWT)	90.12 ± 4.72	**2.31**	**0.029**	92.44 ± 5.3	−1.94	NS	90.5 ± 6.96	0.38	NS
SaO_2_, % (after 6MWT)	80.62 ± 12.38	2.44	NS	83.06 ± 11.24	−1.81	NS	81.25 ± 10.07	0.62	NS
Dyspnea (before 6MWT)	0.81 ± 0.83	**−0.69**	**0.011**	0.12 ± 0.34	− 0.06	NS	0.06 ± 0.25	**− 0.75**	**0.006**
Dyspnea (after 6MWT)	6 ± 2	−1.25	NS	4.75 ± 1.81	0.06	NS	4.81 ± 1.94	−1.19	NS
Fatigue (before 6MWT)	0.5 ± 0.73	−0.38	NS	0.12 ± 0.34	0.19	NS	0.31 ± 0.79	−0.19	NS
Fatigue (after 6MWT)	6.06 ± 1.95	−0.81	NS	5.25 ± 2.35	−0.5	NS	4.75 ± 2.24	**−1.31**	**0.01**
**Body Mass Composition**									
Weight, kg	75.29 ± 19.1	0.69	NS	75.98 ± 18.83	0.55	NS	76.53 ± 19.8	1.24	NS
Body Mass Index, kg/m^2^	25.79 ± 5.78	0.34	NS	26.12 ± 5.53	0.18	NS	26.31 ± 5.96	0.52	NS
Waist-Hip Ratio (WHR)	0.96 ± 0.12	0	NS	0.96 ± 0.12	0.1	NS	0.95 ± 0.13	0.1	NS
Skeletal Muscle Mass, kg	27.54 ± 6.43	0.66	NS	28.2 ± 6.51	0.28	NS	28.48 ± 6.92	0.94	NS
Body Fat Mass, kg	24.54 ± 11.17	−0.1	NS	24.44 ± 10.61	0.1	NS	24.54 ± 10.2	−0.01	NS
Percentage of Body Fat, %	31.32 ± 9.08	−0.19	NS	31.14 ± 8.8	0.01	NS	31.14 ± 7.85	−0.18	NS
Arm Circumference	32.28 ± 5.47	0.22	NS	32.5 ± 5.37	−0.06	NS	32.34 ± 5.36	0.16	NS
Arm Muscle Circumference	25.29 ± 4.11	**0.43**	**0.005**	25.73 ± 4.04	−0.21	NS	25.51 ± 3.99	0.22	NS
**SF-36**									
Physical Functioning	55 ± 28.81	**12.81**	**< 0.001**	67.81 ± 24.76	−3.75	NS	64.06 ± 24.17	9.07	NS
Role Physical	50 ± 29.4	**17.97**	**0.015**	67.97 ± 25.81	−8.59	NS	59.38 ± 26.02	9.38	NS
Bodily Pain	63.38 ± 29.6	6.56	NS	69.94 ± 28.74	3.62	NS	73.56 ± 24.39	10.19	NS
General Health	41.06 ± 24.06	4.19	NS	45.25 ± 18.83	−1.94	NS	43.31 ± 17.37	2.25	NS
Vitality	50.39 ± 24.52	**10.55**	**0.022**	60.94 ± 18.61	−2.34	NS	58.59 ± 21.27	8.2	NS
Social Functioning	66.41 ± 28.77	4.69	NS	71.09 ± 22.69	0.78	NS	71.88 ± 27.2	5.47	NS
Role Emotional	69.27 ± 32.16	**13.02**	**0.029**	82.29 ± 20.61	−4.17	NS	78.13 ± 27.87	8.86	NS
Mental Health	68.75 ± 22.55	0.94	NS	69.69 ± 19.1	−1.56	NS	68.12 ± 18.25	−0.62	NS
Physical Component Summary	40.11 ± 11.35	**4.66**	**0.005**	44.77 ± 9.05	−0.98	NS	43.79 ± 8.13	3.68	NS
Mental Component Summary	47.55 ± 13.33	2.07	NS	49.63 ± 9.27	−0.75	NS	48.88 ± 11.27	1.33	NS
**Fatigue Severity Scale**	4.08 ± 1.6	−0.7	NS	3.38 ± 1.5	0.24	NS	3.61 ± 1.5	−0.47	NS

Values are mean ± standard deviation

6MWD: 6-min walk distance; 6MWT: 6-min walk test; NS: not significant; PEmax, maximal expiratory pressure; PImax, maximal inspiratory pressure; SaO_2_: oxygen saturation; SF-36: 36-Item short form health survey; time 1: measurements before intervention period; time 2: measurements after intervention period; time 3: measurements after observation period

A limited number of adverse events were reported, including excessive fatigue (*n* = 1), and a single incident of a sudden increase in HR during training, which aroused anxiety in the patient (*n* = 1) as the recommended maximum training heart rate was exceeded. Some patients (*n* = 3) reported the common cold, influenza, and chest injury, which were not related to the training program. There were no cases of death in either PAH group.

Adherence to the recommended exercise protocol, which included a minimum of 120 sessions in 24 weeks, was on average 91.88 ± 14.1%. Three participants (18.8%) exercised only 3–4 times per week while nine patients (56.3%) – more than 5 times per week. The average number of training sessions per week was 5.03 ± 1.12; the average number of steps taken during one training session was 4137 ± 1629, and the total number of steps taken within one day, including those taken while training, was 11,341 ± 2845 (the values are the mean of the 6-month intervention period). There were no statistically significant differences between the mean total number of steps taken within one day in the first and last week of intervention (mean at week 1: 11560 ± 3127 vs mean at week 24: 10488 ± 3653), and the mean number of steps taken during a single training session (mean at week 1: 4161 ± 1935 vs mean at week 24: 3929 ± 1301).

Following the intervention period, patients could decide whether they wanted to continue the training program in accordance with the provided protocol. Two patients (13.3%) continued regular training throughout the observation period. Five individuals (33.3%) exercised occasionally. Two patients (13.3%) regularly performed only respiratory muscle training, and provided subjective reports regarding its high effectiveness.

## Discussion

Our study is the first investigation covering a 12-month period that proves both the effectiveness and safety of a simple and low-cost home training program dedicated to patients with PAH. The results of our research demonstrate the beneficial effect of a home-based rehabilitation program on patients’ functional status and quality of life. The effectiveness of the program is comparable or greater than that reported in previously published interventions based mainly on closely supervised rehabilitation in medical facilities. The impact of physical training on functional parameters has been revealed in meta-analyses [12, 25, 26] which demonstrated, inter alia, improvements in 6MWT (6MWD) by 53–72 m, peak VO_2_/kg in the range of 1.5–2.2 ml/min/kg, and workload by 14.9 W [9]. Our study, compared with these analyses, showed an improvement in 6MWD by 71.4 m after the intervention period, revealing equally high effectiveness. In other studies [27–29] evaluating the effectiveness of home-based rehabilitation programs, a significantly less marked effect was achieved (39–48.5 m). Mathai et al. [30] estimated a minimal clinically important difference (MCID) for the 6MWD as the primary outcome measure for patients with PAH proposing the value of approximately 33 m. In our study, twelve patients (75%) surpassed the MCID after the intervention period. The 6-month observation period, which followed the intervention phase, during which the majority of participants did not undertake training, allowed us to observe stabilization of improvement in 6MWD at the level of 57 m. This result is highly satisfactory since it indicates a statistically significant long-term improvement in patients’ health and exceeds the MCID for the population of patients with PAH [30].

Regular exercise training over a long period of intervention led to a statistically significant increase in the resting level of SaO_2._. Regrettably, the effect did not persist in the subsequent six months of observation.

In our study, values of handgrip strength in the PAH group did not differ significantly from those in the control group at the time of inclusion in the study. The training program developed for the purpose of this study did not include exercises with significant resistance [31] due to insufficient evidence regarding the safety of this form of exercise, and a lack of direct medical supervision during home training. Despite the exclusion of typical resistance training from the rehabilitation program, a statistically significant improvement in skeletal muscle strength was achieved, although the effect was not permanent.

The rehabilitation program developed for the purpose of the present study led to an improvement in PImax and PEmax after the intervention period, and an increase in PImax and PEmax after the observation period compared to baseline values. The undertaken respiratory training led to both an improvement in inspiratory muscle strength, as confirmed by previous reports [9, 31–35]), and expiratory muscle strength despite a lack of specific resistance exercises for this group of muscles in the program. This result confirms high effectiveness of exercise training which, in our opinion, should include respiratory muscle resistance training together with exercises mobilizing the chest and diaphragm, and restoring proper function of the respiratory system.

To date, no reports examining the effect of physical training on body composition in patients with PAH have been published in the available literature. In our study, we assessed respiratory muscle and handgrip strength, body composition, and changes in these parameters after a period of cardiac rehabilitation. Body composition analysis using BIA at the recruitment stage of the study showed that there were no statistically significant differences between patients with PAH and healthy controls. Following the 6-month intervention period, there were no statistically significant changes in the investigated parameters, except for an increase in arm muscle circumference. It was found that despite the passage of time and a tendency towards increasing body weight and body mass index (BMI), patients maintained the percentage of body fat (PBF) at a constant level. Additionally, an increase in skeletal muscle mass (SMM), and a statistically significant increase in arm muscle circumference were observed. The beneficial effect of the rehabilitation program was observed not only on muscle function, but also muscle mass despite the fact that typical resistance exercises were not included in the program. These parameters appear to indicate a significant impact of the proposed exercise training program on the increase in skeletal muscle mass, and thus the respiratory muscles. Patient adherence to the rehabilitation program was enhanced by the coordinated efforts of the therapeutic team who, in addition to face-to-face education of patients and their families during follow-up meetings, were in constant telephone contact with patients. Doctors examining study participants during scheduled appointments in the Cardiac Outpatient Clinic were also instructed to monitor both the patients’ condition, and the completion of the self-control diary. Exercising patients were also required to monitor their physical activity based on accelerometry. The support model proved to be highly effective, and the compliance rate of 92% indicated the selection of measures was appropriate. Comparable results have been obtained with other physical training programs in a shorter, 12-week home-based model [28, 29].

During the six-month observation phase, which followed the compulsory intervention period, patients almost completely stopped following the recommended training protocol. Only some of them continued to use the respiratory muscle trainer, and tried to monitor daily physical activity with the use of an accelerometer. Therefore, the lower compliance rate in studies by Babu et al. [27] may be related not only to the etiology of pulmonary hypertension, and the WHO functional class to which the patient belongs at the time of recruitment, but also to a lack of additional tools monitoring patients’ physical activity. On the other hand, decreased frequency of exercise training [27] (3 sessions a week compared to 5–6 sessions in other studies [28, 29]), may give patients increased freedom to forego training sessions. These differences lessen the possibility of comparing the results of our study with those reported in the aforementioned paper. There may be some possible limitations in this study. The first is the small sample size caused by the low incidence of PAH in the general population (estimated prevalence and annual incidence of PAH in Poland – 30.8/mln and 5.2/mln, respectively [36]) and exclusion of patients unable to follow the recommended training program. A review of the available literature indicates that a small sample size has been listed as a limitation in the vast majority of published studies, which explains a small number of randomized clinical trials in PAH. In multiple studies evaluating the effectiveness of rehabilitation in patients with pulmonary hypertension, the number of patients in the intervention group ranged from 12 to 30. The second limitation concerns the model of monitoring adherence to therapeutic recommendations which was based on self-reports of patients supported and supervised by a carer/family member, and weekly telephone verification by the research team. Despite the fact that this type of method is commonly used in long-term cardiac rehabilitation, the use of telemonitoring will be a standard way of healthcare delivery in the future. The third limitation is connected with the ongoing COVID-19 pandemic which made evaluation of the control group one year after patient inclusion in the study impossible. Inability to compare the functional status of patients in the PAH intervention group with those who did not undertake training after 12 months of study enrollment limits the ability to determine the scale of disease-related physical impairment over time. Due to the dynamically developing pandemic, and the introduction of a national lockdown at the time, patients from the control group, who did not benefit from joining the study as they did not experience the positive effects of rehabilitation, showed very limited motivation to participate in the final evaluation. Due to the fact that PAH as a concomitant disease may have affected the severity of a potential COVID-19 infection, patients declined to attend scheduled outpatient appointments since they associated healthcare settings with an increased risk of contracting the disease. Therefore, taking into consideration the benefit-risk ratio, re-evaluation the control group was not insisted upon. By contrast, despite the risks associated with the current pandemic, participants from the intervention group expressed the need to undergo a set of tests designed to demonstrate the positive impact of the rehabilitation program on their functional status and physical fitness.

In summary, the present study provides key data on the feasibility of the developed home-based exercise training program and offers hope for physiotherapeutic care for patients with PAH who do not have access to other forms of rehabilitation. The results of our study prove that the home-based exercise program developed by us is safe, effective and acceptable to patients with PAH, which encourages its implementation in centers which do not provide inpatient or outpatient rehabilitation.

## Conclusions

Home-based rehabilitation program is an entirely safe form of rehabilitation for stable patients with PAH who can be supervised by a family member/caregiver.

Home-based rehabilitation significantly improves functional parameters, quality of life and physical fitness.

Respiratory muscle strength and 6MWD remain increased even six months after training cessation.

A lower level of resting dyspnea persists six months after training cessation.

The effects of increased hand grip strength, increased resting saturation, and improved quality of life are not sustained after training cessation.

## Data Availability

Datasets used and/or analyzed during the study are available from the corresponding author on reasonable request.

## Abbreviations

6MWD: 6-min walk distance
6 MWT: 6-min walk test
ASEP: American Society of Exercise Physiologists
ATS: American Thoracic Society
BIA: bioimpedance analysis
BMI: body mass index
CO: cardiac output
CPET: cardiopulmonary exercise testing
ERS: European Respiratory Society
ESC: European Cardiology Society
FSS: Fatigue Severity Scale
HGS: hand grip strength
HR: heart rate
HRQOL: health-related quality of life
MCID: minimal clinically important difference
mPAP: mean pulmonary arterial pressure
NT-proBNP: N-terminal brain natriuretic peptide
PAH: pulmonary arterial hypertension
PAWP: pulmonary artery wedge pressure
PBF: percentage of body fat
PEmax: maximum expiratory pressure
PImax: maximum inspiratory pressure
PVR: pulmonary vascular resistance
RHC: right-heart catheterization
SaO_2_: oxygen saturation
SF-36: 36-Item Short Form Health Survey
SMM: skeletal muscle mass
TAPSE: tricuspid annular plane systolic excursion
TTE: transthoracic echocardiography
VCO_2_: carbon dioxide production
VE: ventilation
VO_2_: oxygen consumption
WHO-FC: World Health Organization Functional Class
WU: Wood units

## References

[1] Simonneau G, Montani D, Celermajer DS, Denton CP, Gatzoulis MA, Krowka M, Williams PG, Souza R (2019). Haemodynamic definitions and updated clinical classification of pulmonary hypertension. Eur Respir J.

[2] Condon DF, Nickel NP, Anderson R, Mirza S, de Jesus Perez VA. The 6th World Symposium on Pulmonary Hypertension: what's old is new. F1000Res. 2019;8:F1000 Faculty Rev-888. 10.12688/f1000research.18811.1 PMC6584967 31249672

[3] Eichstaedt CA, Benjamin N, Xanthouli P, Marra AM, Grünig E (2019). The role of rehabilitation in patients with pulmonary arterial hypertension. Curr Opin Pulm Med.

[4] Dean BB, Saundankar V, Stafkey-Mailey D, Anguiano RH, Nelsen AC, Gordon K, Classi P (2020). Medication adherence and healthcare costs among patients with pulmonary arterial hypertension treated with Oral Prostacyclins: a retrospective cohort study. Drugs Real World Outcomes.

[5] Benza RL, Gomberg-Maitland M, Elliott CG, Farber HW, Foreman AJ, Frost AE, McGoon MD, Pasta DJ, Selej M, Burger CD, Frantz RP (2019). Predicting survival in patients with pulmonary arterial hypertension: the REVEAL risk score calculator 2.0 and comparison with ESC/ERS-based risk assessment strategies. Chest..

[6] Morris ZV, Chin LMK, Chan L, Guccione AA, Ahmad A, Keyser RE (2020). Cardiopulmonary exercise test indices of respiratory buffering before and after aerobic exercise training in women with pulmonary hypertension: differentiation by magnitudes of change in six-minute walk test performance. Respir Med.

[7] Sisniega C, Zayas N, Pulido T (2019). Advances in medical therapy for pulmonary arterial hypertension. Curr Opin Cardiol.

[8] Galiè N, Humbert M, Vachiery JL, Gibbs S, Lang I, Torbicki A, Simonneau G, Peacock A, Vonk Noordegraaf A, Beghetti M, Ghofrani A, Gomez Sanchez MA, Hansmann G, Klepetko W, Lancellotti P, Matucci M, McDonagh T, Pierard LA, Trindade PT, Zompatori M, Hoeper M, ESC Scientific Document Group (2016). 2015 ESC/ERS guidelines for the diagnosis and treatment of pulmonary hypertension: the joint task force for the diagnosis and treatment of pulmonary hypertension of the European Society of Cardiology (ESC) and the European Respiratory Society (ERS): endorsed by: Association for European Paediatric and Congenital Cardiology (AEPC), International Society for Heart and Lung Transplantation (ISHLT). Eur Heart J.

[9] Grünig E, Eichstaedt C, Barberà JA, Benjamin N, Blanco I, Bossone E, Cittadini A, Coghlan G, Corris P, D'Alto M, D'Andrea A, Delcroix M, de Man F, Gaine S, Ghio S, Gibbs S, Gumbiene L, Howard LS, Johnson M, Jurevičienė E, Kiely DG, Kovacs G, MacKenzie A, Marra AM, McCaffrey N, McCaughey P, Naeije R, Olschewski H, Pepke-Zaba J, Reis A, Santos M, Saxer S, Tulloh RM, Ulrich S, Vonk Noordegraaf A, Peacock AJ (2019). ERS statement on exercise training and rehabilitation in patients with severe chronic pulmonary hypertension. Eur Respir J.

[10] Johnson MK, Peacock AJ (2019). Treating pulmonary arterial hypertension with exercise: the role of rehabilitative medicine. Adv Pulm Hyperten.

[11] Koudstaal T, Wapenaar M, van Ranst D, Beesems R, van den Toorn L, van den Bosch A, Chandoesing P, Boomars K (2019). The effects of a 10-wk outpatient pulmonary rehabilitation program on exercise performance, muscle strength, soluble biomarkers, and quality of life in patients with pulmonary hypertension. J Cardiopulm Rehabil Prev.

[12] Glöckl R, Schneeberger T, Boeselt T, Kenn K, Koczulla AR, Held M, Oberhoffer R, Halle M (2019). Körperliches training bei pulmonaler Hypertonie – ein systematisches review mit Metaanalyse [exercise training in patients with pulmonary hypertension: a systematic review and meta-analysis]. Pneumologie..

[13] Ozemek C, Berry MJ, Arena R (2019). A review of exercise interventions in pulmonary arterial hypertension and recommendations for rehabilitation programing. J Cardiopulm Rehabil Prev..

[14] Vallerand JR, Weatherald J, Laveneziana P (2019). Pulmonary hypertension and exercise. Clin Chest Med.

[15] Dalla Vecchia LA, Bussotti M (2018). Exercise training in pulmonary arterial hypertension. J Thorac Dis..

[16] Nogueira-Ferreira R, Moreira-Gonçalves D, Santos M, Trindade F, Ferreira R, Henriques-Coelho T (2018). Mechanisms underlying the impact of exercise training in pulmonary arterial hypertension. Respir Med.

[17] Riou M, Pizzimenti M, Enache I, Charloux A, Canuet M, Andres E, Talha S, Meyer A, Geny B (2020). Skeletal and respiratory muscle dysfunctions in pulmonary arterial hypertension. J Clin Med.

[18] Lang RM, Badano LP, Mor-Avi V (2015). Recommendations for cardiac chamber quantification by echocardiography in adults: an update from the American Society of Echocardiography and the European Association of Cardiovascular Imaging. J Am Soc Echocardiogr.

[19] Rudski LG, Lai WW, Afilalo J (2010). Guidelines for the echocardiographic assessment of the right heart in adults: a report from the American Society of Echocardiography endorsed by the European Association of Echocardiography, a registered branch of the European Society of Cardiology, and the Canadian Society of Echocardiography. J Am Soc Echocardiogr.

[20] American Thoracic Society; American College of Chest Physicians (2003). ATS/ACCP statement on cardiopulmonary exercise testing. Am J Respir Crit Care Med.

[21] ATS Committee on Proficiency Standards for Clinical Pulmonary Function Laboratories (2002). ATS statement: guidelines for the six-minute walk test. Am J Respir Crit Care Med.

[22] Brown LE, Weir JP (2001). ASEP procedures recommendation I: accurate assessment of muscular strength and power. JEPonline..

[23] American Thoracic Society/European Respiratory Society (2002). ATS/ERS statement on respiratory muscle testing. Am J Respir Crit Care Med.

[24] Laveneziana P, Albuquerque A, Aliverti A, Babb T, Barreiro E, Dres M, Dubé BP, Fauroux B, Gea J, Guenette JA, Hudson AL, Kabitz HJ, Laghi F, Langer D, Luo YM, Neder JA, O'Donnell D, Polkey MI, Rabinovich RA, Rossi A, Series F, Similowski T, Spengler C, Vogiatzis I, Verges S (2019). ERS statement on respiratory muscle testing at rest and during exercise. Eur Respir J.

[25] Zeng X, Chen H, Ruan H, Ye X, Li J, Hong C (2020). Effectiveness and safety of exercise training and rehabilitation in pulmonary hypertension: a systematic review and meta-analysis. J Thorac Dis.

[26] Morris NR, Kermeen FD, Holland AE (2017). Exercise-based rehabilitation programmes for pulmonary hypertension. Cochrane Database Syst Rev.

[27] Babu AS, Padmakumar R, Nayak K, Shetty R, Mohapatra AK, Maiya AG (2019). Effects of home-based exercise training on functional outcomes and quality of life in patients with pulmonary hypertension: a randomized clinical trial. Indian Heart J.

[28] Butāne L, Šmite D, Šablinskis M, Skride A (2019). Individualized home-based exercise program for idiopathic pulmonary arterial hypertension patients: a preliminary study. Cor Vasa.

[29] Brown MB, Kempf A, Collins CM, Long GM, Owens M, Gupta S, Hellman Y, Wong V, Farber M, Lahm T (2018). A prescribed walking regimen plus arginine supplementation improves function and quality of life for patients with pulmonary arterial hypertension: a pilot study. Pulm Circ.

[30] Mathai SC, Puhan MA, Lam D, Wise RA (2012). The minimal important difference in the 6-minute walk test for patients with pulmonary arterial hypertension. Am J Respir Crit Care Med.

[31] González-Saiz L, Fiuza-Luces C, Sanchis-Gomar F, Santos-Lozano A, Quezada-Loaiza CA, Flox-Camacho A, Munguía-Izquierdo D, Ara I, Santalla A, Morán M, Sanz-Ayan P, Escribano-Subías P, Lucia A (2017). Benefits of skeletal-muscle exercise training in pulmonary arterial hypertension: the WHOLEi+12 trial. Int J Cardiol.

[32] Aslan GK, Akıncı B, Yeldan I, Okumus G (2020). A randomized controlled trial on inspiratory muscle training in pulmonary hypertension: effects on respiratory functions, functional exercise capacity, physical activity, and quality of life. Heart Lung.

[33] Aslan GK, Akinci B, Yeldan I, Okumus G (2018). Respiratory muscle strength in patients with pulmonary hypertension: the relationship with exercise capacity, physical activity level, and quality of life. Clin Respir J.

[34] Saglam M, Arikan H, Vardar-Yagli N, Calik-Kutukcu E, Inal-Ince D, Savci S, Akdogan A, Yokusoglu M, Kaya EB, Tokgozoglu L (2015). Inspiratory muscle training in pulmonary arterial hypertension. J Cardiopulm Rehabil Prev..

[35] Kabitz HJ, Bremer HC, Schwoerer A, Sonntag F, Walterspacher S, Walker DJ, Ehlken N, Staehler G, Windisch W, Grünig E (2014). The combination of exercise and respiratory training improves respiratory muscle function in pulmonary hypertension. Lung..

[36] Kopeć G, Kurzyna M, Mroczek E, Chrzanowski Ł, Mularek-Kubzdela T, Skoczylas I, Kuśmierczyk B, Pruszczyk P, Błaszczak P, Lewicka E, Karasek D, Mizia-Stec K, Tomaszewski M, Jacheć W, Ptaszyńska-Kopczyńska K, Peregud-Pogorzelska M, Doboszyńska A, Pawlak A, Gąsior Z, Zabłocka W, Ryczek R, Widejko-Pietkiewicz K, Waligóra M, Darocha S, Furdal M, Ciurzyński M, Kasprzak JD, Grabka M, Kamiński K, Hoffman P, Podolec P, Torbicki A (2020). Characterization of patients with pulmonary arterial hypertension: data from the polish registry of pulmonary hypertension (BNP-PL). J Clin Med.

